# A short add-on sleep intervention in the rehabilitation of individuals with acquired brain injury: A randomized controlled trial

**DOI:** 10.3233/NRE-230139

**Published:** 2023-11-14

**Authors:** Louise Pilon, Nikita F. Frankenmolen, Janna van der Zijp, Roy P.C. Kessels, Dirk Bertens

**Affiliations:** a Donders Institute for Brain, Cognition and Behaviour, Radboud University, Nijmegen, The Netherlands; b Vincent van Gogh Institute for Psychiatry, Venray, The Netherlands; c Rehabilitation Medical Centre Klimmendaal, Arnhem, The Netherlands

**Keywords:** Acquired brain injury, stroke, traumatic brain injury, sleep disturbances, RCT, neuropsychological rehabilitation

## Abstract

**BACKGROUND:**

Sleep disturbances are common after acquired brain injury (ABI) and have a negative impact on functioning.

**OBJECTIVE:**

This study examines whether a short add-on therapy for sleep disturbances in individuals with ABI is effective in addition to rehabilitation treatment as usual.

**METHODS:**

In the randomized-controlled study, 54 adults with ABI and self-reported sleep disturbances receiving outpatient rehabilitation services were randomized in two groups: one receiving a sleep intervention (based on cognitive behavioural therapy for insomnia (CBT-I)) in addition to their rehabilitation treatment (CBT-I + TAU group) and one receiving treatment as usual (TAU). The primary outcome was sleep quality, measured with the Pittsburgh Sleep Quality Index (PSQI). Secondary outcomes included measures of anxiety, depression, fatigue and dysfunctional beliefs and attitudes about sleep.

**RESULTS:**

The short add-on sleep therapy resulted in improvements in sleep quality in the CBT-I + TAU group as compared to the TAU group (ES = 0.924). Furthermore, the CBT-I + TAU group reported less dysfunctional beliefs and attitudes about sleep and were better able to cope with fatigue compared to the TAU group.

**CONCLUSIONS:**

The application of this short add-on sleep intervention could be implemented in neuropsychological rehabilitation settings.

## 1 Introduction

Individuals with acquired brain injury (ABI) often have a wide range of impairments which may result in long-term difficulties in everyday life (Bradt et al., 2010). Persons with ABI often experience problems with motor function, cognition (e.g. thinking and memory), emotional disturbances, fatigue and somatic complaints (Belmont et al., 2006; Fleminger & Ponsford, 2005; Larson, 2018; Prince & Bruhns, 2017; Staub & Bogousslavsky, 2001; Vallat-Azouvi et al., 2018). In addition, sleep disturbances are also common in people with ABI and often have a negative impact on daily functioning (Biajar, 2017). More than half of individuals with ABI report sleep-related problems (Campos TF, 2005; Mathias & Alvaro, 2012). Many of these individuals who report sleep disturbances fulfill the DSM-V criteria for insomnia disorder; up to a third of individuals with ABI (including stroke and TBI) are diagnosed with insomnia disorder (Leppavuori et al., 2002; Ouellet et al., 2015), while the incidence of insomnia in the general population is approximately 10% (Ohayon, 2002).

Sleep disturbances negatively affect health, resulting in a lower quality of life, decline in cognitive abilities and a higher risk of mood disorders (Medic et al., 2017). Following ABI, sleep disturbances can exacerbate other injury-related symptoms (e.g., cognitive functioning) and have a negative impact on recovery (Sandsmark et al., 2017; Zuzuárregui JRP, 2018). Furthermore, sleep disturbances following ABI are associated with increased feelings of anxiety and depression (Parcell et al., 2008) and a lower quality of life (Cantor et al., 2008). There is also evidence for limitations in daily functioning in several other domains, such as societal participation or physical activities (Fogelberg et al., 2012). As a result, the consequences of sleep disturbances delay and hinder the rehabilitation process (Sandsmark et al., 2017; Zuzuárregui JRP, 2018). The high prevalence of self-reported sleep disturbances and their negative outcomes stress the need for developing effective sleep interventions for individuals with ABI.

According to the European Sleep Research Society (ESRS), the preferred treatment of sleep disturbances in the healthy population consists of non-pharmacological treatments, such as cognitive behavioural therapy for insomnia (CBT-I) (Riemann et al., 2017). CBT-I is an evidence-based approach for treatment of sleep disturbances, which aims to alter inadequate beliefs and behaviour leading to dysfunctional sleep-wake rhythms (Riemann et al., 2017). It should be noted that pharmacological treatments are also recommended for the treatment of insomnia, but only as a short-term therapy or when CBT-I is ineffective or unavailable (Riemann et al., 2017; Saddicha, 2010). The main components of CBT-I include psychoeducation, improving sleep hygiene, sleep restriction and stimulus control (Riemann et al., 2017). Advantages of a (cognitive)-behavioural approach over a pharmacological one include strong empirical evidence regarding the long-term effectiveness in the absence of a risk of dependence or other negative side effects (Baglioni et al., 2020; Thomas & Greenwald, 2018).

While studies in healthy individuals have thus provided clear evidence for CBT-I as the treatment of choice for sleep disturbances, there is to date limited evidence for the effectiveness of CBT-I following ABI. Based on several systematic reviews, CBT interventions are most promising for improving sleep following ABI (Ford et al., 2020; Herron et al., 2018; Nguyen et al., 2019; Pilon et al., 2021; Theadom et al., 2018; Thomas & Greenwald, 2018; Ymer et al., 2021). Although these CBT-I interventions for ABI roughly include the same components as CBT-I for the general population (such as stimulus control and sleep restriction), they usually have a larger number of sessions (e.g. 7 or 8 sessions) and in administration form (e.g., face to face treatment with trained therapists, telehealth or a combination of both) (Herron et al., 2018; Nguyen et al., 2019; Theadom et al., 2018; Ymer et al., 2021). A problem with the existing CBT-I studies in ABI patients is that the interventions have been developed and studied in a monodisciplinary context (Nguyen et al., 2019; Ymer et al., 2021), which requires trained CBT therapists, making them less suitable for a multidisciplinary rehabilitation setting. Furthermore, CBT-I interventions overlap with rehabilitation interventions targeting fatigue or stress, such as relaxation methods, pacing or graded activity (Stubberud et al., 2019). Moreover, specific sleep-related advises is often not integrated in current rehabilitation programs of individuals with ABI.

The aim of the present study is to examine whether a short add-on therapy for sleep disturbances in individuals with ABI is effective in an outpatient rehabilitation setting. The add-on sleep therapy was developed based on CBT-I components, which have shown to be effective in previous studies (Herron et al., 2018; Nguyen et al., 2019; Ymer et al., 2021). However, the add-on intervention is delivered in a shorter amount of time (4 sessions in a period of 6 weeks) and has less overlap with standard rehabilitation interventions targeting fatigue. The add-on therapy was provided by trained cognitive rehabilitation therapists (e.g. occupational therapists). Thus, the add-on sleep therapy is a short variant of CBT-I which is in addition to rehabilitation treatment as usual. This add-on sleep therapy focuses solely on sleep behaviour at night and consists of psychoeducation, improvement of sleep hygiene, stimulus control, and an adapted form of sleep restriction. The add-on therapy has already been implemented in the participating rehabilitation, with anecdotal positive responses from both patients and care professionals, but a controlled study on its efficacy has not been performed so far.

The main hypothesis of the present study is that the sleep quality significantly improves in the group who received the short add-on sleep therapy in addition to treatment as usual (CBT-I + TAU) compared to the group that received rehabilitation treatment as usual only (TAU only). We also hypothesize that the treatment group (CBT-I + TAU) will demonstrate less fatigue and dysfunctional beliefs and attitudes about sleep, and less feelings of anxiety and depression. In addition to a group analysis, the effectiveness of the short add-on sleep therapy will also be studied at the individual level. Reliable change indices (RCI) for the primary outcome measure (reported sleep quality) will be calculated for each patient to determine whether the short add-on therapy resulted in clinically significant changes in sleep quality. The short add-on therapy may lead to a valuable contribution to neuropsychological rehabilitation in clinical practice.

## 2 Method

### 2.1 Participants

Participants were recruited from the outpatient department of the Rehabilitation Medical Centre Klimmendaal in Arnhem, The Netherlands. To be eligible for inclusion, participants (1) had to have an acquired brain injury (≥3 months post-injury), (2) had self-reported sleep problems which are associated with disability in daily life (not confirmed by a self-reported questionnaire measuring sleep disturbances) (3) were aged between 18 and 75 and (4) received outpatient rehabilitation services. Exclusion criteria were (1) severe brain damage, (2) neurodegenerative disorders (e.g., Parkinson, Multiple Sclerosis), (3) were diagnosed with a sleep disorder prior to injury (e.g., obstructive sleep apnoea, restless legs syndrome), (4) alcohol or drug abuse or dependence, (5) had serious psychopathology (e.g., risk for psychosis) or expressed suicidal ideations and (6) recently started taking sleep medication (less than a month ago). All participants voluntarily participated in this study. Sleep disturbances refer to sleep problems that occur at night and are characterized by the inability to initiate and maintain sleep and are associated with fatigue during daytime.

For the calculation of the required sample size, G*Power for ANOVA was used (Faul et al., 2009). An estimation of the correlation among repeated measures was used to determine the required sample size. A previous study determined a correlation among repeated measures of 0.68 for PSQI scores with a one-year interval in 610 healthy individuals (Knutson et al., 2006). However, there is no data available about the correlation among repeated measures in an ABI population. Therefore, based on the study of (Knutson et al., 2006), we used an estimation of the correlation among repeated measures of 0.60. A recent meta-analysis demonstrated that the effect sizes of CBT-I were medium to large in normal populations as compared to non-active control groups (van der Zweerde et al., 2019). We preferred to be more conservative and expect a somewhat smaller than medium effect size *(effect size f;* 0.2) in an ABI population and because of the comparison with an active control group (e.g. treatment as usual). Also, the present study used a shorter add-on intervention compared to the existing CBT-I studies. A total sample size of 42 participants that completed the intervention was required to detect an effect size *f* of 0.2 with a sufficient power of 0.80 at a significance level of 0.05.

The present study was registered at The Dutch Trial Register; registration number NL9368; https://onderzoekmetmensen.nl/nl/trial/24225) and approved by the local institutional review board of Klimmendaal Rehabilitation Centre prior to the beginning of the study. Demographic variables (e.g. sex, age, education level) and injury-related details were collected at baseline. Education level was determined using a 7-point rating scale, with 1 indicating “*less then primary school*” and 7 “*university degree*” according to the Dutch educational classification of Verhage (1964).

### 2.2 Outcome measures

For evaluation of the effects of the short add-on sleep therapy, validated Dutch versions of four self-reported questionnaires were administered, including the Pittsburg Sleep Quality Index (PSQI), the Dutch Multi-Factor Fatigue Scale (DMFS), the Hospital Anxiety and Depression Scale (HADS) and the Dysfunctional Beliefs and Attitudes about Sleep Scale brief version (DBAS-16). All self-report questionnaires were administered at baseline and post-treatment.

The primary outcome measure was the self-report Pittsburg Sleep Quality Index (PSQI) questionnaire (Buysse et al., 1989). The PSQI is widely used in clinical practice assessing subjective sleep quality, duration of falling asleep, total sleep time and sleep efficiency (SE) (SE = total sleep time / total time in bed×100%) in the previous month (Backhaus et al., 2002). This self-report instrument is grouped into 7 subscales (sleep quality, sleep latency, sleep duration, sleep efficiency, sleep disturbances, sleep medication, and daytime dysfunction) that are rated on a 3-point scale. The final PSQI score ranges from 0–21, with a higher score indicating a worse sleep quality. The established cut-off score of >5 points on the PSQI mean score is used to indicate clinical sleep complaints (Buysse et al., 1989).

Secondary outcome measures included the DMFS, the HADS and the DBAS-16. The Dutch Multi-Factor Fatigue Scale (DMFS) is a self-report questionnaire and considered to be a subjective measure of fatigue (Visser-Keizer et al., 2015). The DMFS consists of 38 items that are rated on a 5-point Likert-scale, with 1 (*totally unagreed*) to 5 (*totally agree*). Nine items are negatively phrased and were be therefore reverse coded. The DMFS consists of 5 subscales, that is impact of fatigue, mental fatigue, signs and direct consequences of fatigue, physical fatigue and coping with fatigue. Higher scores represent more severe (consequences of) fatigue or dysfunctional coping with fatigue.

Anxiety and depressive symptoms were examined using the Hospital Anxiety and Depression Scale (HADS) (Zigmond & Snaith, 1983). The HADS is a self-rating questionnaire consisting of 14 multiple-choice items that are rated on a 4-point Likert scale. Total scores range from 0–21, with higher scores indicating more anxiety- and depression-related complaints. The HADS consists of 2 subscales, namely anxiety (HADS-A) and depression (HADS-D). Both subscales consist of 7 items.

The Dysfunctional Beliefs and Attitudes about Sleep Scale brief version (DBAS-16) was administered to assess dysfunctional beliefs and attitudes about sleep (Morin, 1993). This self-reported questionnaire consists of 16 items. Items are rated on a 5-point Likert scale (2-4-6-8-10) ranging from *strongly disagree* (2) to *strongly agree* (10). The DBAS-16 consists of 4 subscales, namely perceived consequences of insomnia, worry/helplessness of insomnia, sleep expectations and medication. An average total score is calculated by summing up all items and dividing this score by 16. A higher total score reflects more dysfunctional beliefs and attitudes about sleep.

### 2.3 Intervention

The add-on sleep intervention comprised four sessions over six weeks and is based on CBT-I principles. The sleep intervention was added to the regular rehabilitation program, which varied between individuals based on their rehabilitation goals. Thus, the add-on sleep intervention is a short variant of CBT-I which is in addition to the rehabilitation treatment as usual. All therapists were trained in CBT-I. Furthermore, individual supervision by an experienced CBT-I therapist was available during the study. During the COVID-19 pandemic lockdown in 2020, treatment sessions were delivered online through video calls and assessments were sent by post.

The first session started with the baseline assessment. During a structured interview, participants were asked to address their sleep-related problems. Subsequently, a daily homework assignment was given to the participants that involved completing a sleep-wake diary for one week. This sleep-wake diary provided information about several (sleep) parameters, including sleep duration, time spent in bed, wake time, sleep quality, nighttime activities, caffeinated drinks and alcohol consumption.

The second session (one week later) consisted of psychoeducation about sleep and recommendations were made to change sleep behaviour. Psychoeducation and recommendations were somewhat personalized based on information gathered from the sleep assessment (interview, questionnaires and sleep diary). Behavioural techniques consisted of discussing sleep hygiene, such as caffeine intake and activities before bedtime, stimulus control and sleep restriction. Stimulus control aims to improve the association between bed and sleep-behaviour by solely using the bed for sleeping (and not for other activities such as reading or watching TV). In addition, an adapted form of sleep restriction was used in which participants were instructed to shorten the time spent in bed to a maximum of 7 hours in order to consolidate sleep. It is important to note that regular CBT-I treatments instruct participants to spent only 5 hours in bed. Since research demonstrated that individuals with ABI need more sleep as compared to individuals without ABI (Baglioni et al., 2016; Imbach et al., 2016), the sleep restriction was adapted to the needs of the individual participants with ABI. Furthermore, the adapted form of sleep restriction allows participants to increase their sleep structure, which also prevents them from spending too much time in bed with ineffective sleep. Participants received all these recommendations at the same time in the same session and were encouraged to follow these strictly for the following two weeks.

After two weeks, the third session took place in which the adjustments in sleep behaviour and its effect on sleep quality were evaluated and, if necessary, extended or adapted. Possible additional elements contained relaxation exercises, strategies to cope with worrying (e.g., ‘worry postponement’) or increasing physical activities during daytime. Participants were instructed to maintain their changes in sleep behaviour for another two weeks.

During the final session, which took place two weeks after the third session, changes in sleep behaviour and its impact on sleep were evaluated. Furthermore, the therapist and participant jointly developed a relapse prevention plan which also included an overview of the most important sleep recommendations. The session ended with the post-treatment assessment (T6: PSQI, HADS, DMFS, DBAS-16). Throughout the treatment, participants were encouraged to follow the program strictly to develop a regular sleep pattern. A summary of the content of the sleep intervention is presented in Table 1.

**Table 1:** Summary of the short add-on sleep therapy

Session	Content
1	Addressing sleep-related problems
	Sleep-diary
2	Psycho-education
	Sleep hygiene
	Stimulus control
	Sleep restriction
3	Evaluation (and adaptation) of sleep behaviour
4	Evaluation of sleep behaviour
	Relapse prevention plan

### 2.4 Procedure

This randomized controlled trial was conducted between September 2018 and February 2022, with the first patient included on 10 September 2018. Potential participants were referred by their neuropsychologists in the first weeks of their rehabilitation program. Neuropsychologists screened possible participants for inclusion and exclusion criteria and gave information about the study. All ABI patients who met the inclusion criteria were able to receive the add-on sleep therapy, regardless of their participation in the study. When participants were interested in participation in the study, the researcher (LP) was informed. A final check on inclusion- and exclusion criteria was performed by the researcher. After obtaining the signed informed consents, participants were randomly allocated to either the CBT-I + TAU or TAU only group using a computer-generated randomization program (Research Randomizer; https://www.randomizer.org). A researcher not involved in the inclusion of the study generated the randomization. Participants in the TAU only group were told that they would receive the sleep intervention after the six-week waiting period. Both groups received a pre- and post-assessment. Participants in the CBT-I + TAU group completed the assessments pre-treatment (T0) and post-treatment (T6). Participants in the TAU only group were assessed pre-waiting period (T0) and post-waiting period (T6). They subsequently received the sleep intervention in the context of providing optimal rehabilitation services.

The CBT-I + TAU group started with the six-week sleep intervention, in addition to their rehabilitation treatment as usual. Patients in the TAU only group were placed on a wait-list for six weeks, while receiving rehabilitation treatment as usual (based on indication, which consisted of physiotherapy, occupational therapy, neuropsychological treatment, speech therapy). Demographic and injury-related information was obtained and the self-report measures (PSQI, HADS, DMFS, DBAS-16) were filled in (T0 for the treatment group) during the first session. Note that results of the baseline measures were not discussed with the participants during the first session. At the end of the sleep intervention, pre-and post- assessments were discussed with patients during the last session.

A follow-up measurement took place in the CBT-I + TAU group to examine whether the effects of the add-on sleep intervention were maintained. The CBT-I + TAU group completed the follow-up assessment 3 months after completing the intervention. Note that the follow-up assessment was limited to the CBT-I + TAU group, as the TAU only group also received the intervention following the 6-week waiting period.

Assessments for the CBT-I + TAU group were built in the treatment sessions, with T0 in the first session and T6 after six weeks in the fourth session and administered by the therapists. At the start of the waiting period, a baseline assessment (T0) was administered by one of the researchers. T6 was carried out in the first session of the intervention. Patients and therapists were aware of the group allocation as the CBT-I + TAU group received the therapy in addition to their rehabilitation treatment as usual, while the TAU only group received the intervention after six weeks. The researcher was not blinded, since her role was to communicate with the planning staff to ensure patients started the intervention at the appropriate timepoint. A CONSORT flow diagram showing the content of the interventions is presented in Fig. 2.

### 2.5 Statistical analyses

For all variables the assumption of normality was checked and corrected for, if necessary. IBM SPSS 27 was used for the statistical analyses (IBM Corp., Armonk, USA). Demographic and baseline data of the two groups were analyzed using two-tailed independent *t* tests to ensure that there were no significant differences in scores on the self-reported measures between two groups at baseline.

To determine the efficacy of the short add-on therapy, per protocol analyses were performed (see The Dutch Trial Register; registration number NL9368; https://onderzoekmetmensen.nl/nl/trial/24225). A two-way (between-subjects factor: treatment vs. wait-list control group; within subjects factor; baseline vs. post-treatment) analysis of variance with repeated measures (Repeated Measures ANOVA) was used to examine group-by-time interactions for scores on the primary outcome measure, that is, the PSQI. The same analysis was used for the secondary outcomes (HADS, subscales of the DMFS, DBAS-16) to evaluate anxiety and mood symptoms, fatigue and dysfunctional beliefs and attitudes about sleep.

Individual-level change scores were calculated using Reliable Change Indices (RCI; (Jacobson & Truax, 1991). The analysis of RCI specifies whether an individual’s change during pre- and post-treatment can be attributed to the add-on therapy (de Souza & de Paula, 2015). Here, we used the method of Jacobson & Truax (1991) for calculating the RCIs. The standard deviation (SD) based on the baseline data of the PSQI was computed for the included participants (N = 53). This proportion was calculated for participants who completed both baseline assessment and post-assessment. For each participant, the difference between the post-assessment and baseline assessment was calculated. A total inclusion of 53 participants with a SD of 3.0 for baseline PSQI scores, a change of 3.21 points was calculated as reliable change. When RCI is higher than 3.21, this indicates a reliable change at a 95% confidence interval (CI). To determine whether an observed individual’s change is reliable, T0 and T6 scores in both groups were used respectively as pre- and posttreatment. In addition, the clinical cut-off score for insomnia (>5) was used to evaluate at post-treatment the percentage of participants that did not fulfill the clinical cut-off score for insomnia. Effect sizes were computed (Cohen’s *d*) and interpreted as small (≥0.2) medium ≥0.5 or large (≥0.8) (Cohen, 2013). Alpha was set at 0.05 for all analyses.

In order to assess whether the effects of the add-on sleep intervention were maintained from post-treatment to 3-month follow-up, paired sample *t*-tests were used to compare post-treatment and 3-month follow-up mean values of the PSQI measure in the add-on group only.

## 3 Results

**Fig. 1:**
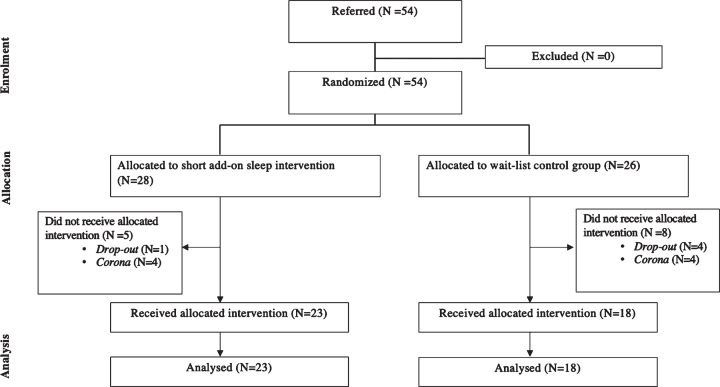
Flow diagram according to CONSORT.

A total of 54 participants were included in the clinical trial after written informed consent was collected. A total of 41 individuals completed this study. Eight participants withdrew before or during the intervention (four participants dropped out during and because of the COVID-19 lockdown and four post-assessments were not returned). Five participants dropped out before the post-treatment assessment (reasons: a spontaneous improvement in sleep disturbances, lack of time, or being preoccupied with current stressful life events). A CONSORT diagram showing the flow of participants through the study phases is presented in Fig. 1. Table 2 shows the demographic characteristics of the study population.

**Fig. 2:**
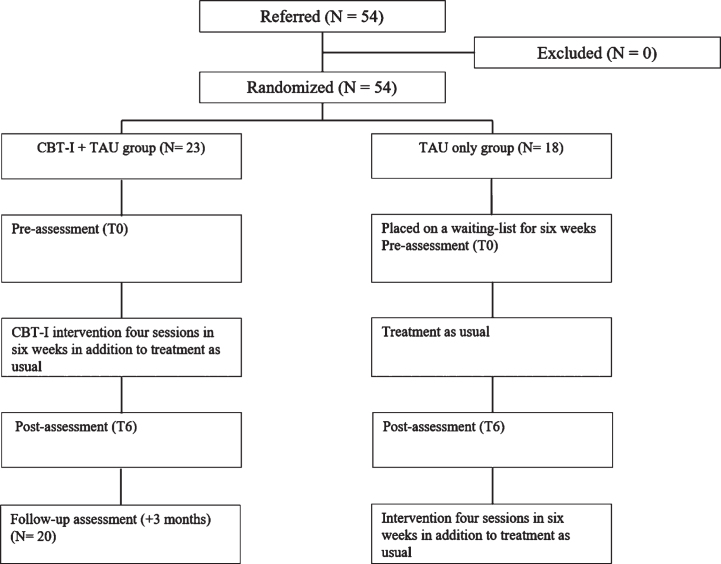
Flowchart of the patients progress through the study phases.

**Table 2:** Baseline and clinical characteristics

	CBT-I + TAU group	TAU only group	*p-*value
	M (SD)	M (SD)
N	23	18
Sex (m/f)	12/11	12/6	0.274
Age (years)	47.74 (12.15)	46.83 (12.98)	0.819
Time since injury (months)	46.13 (20.84)	70.44 (40.65)	0.124
Education level	5.61 (.84)	5.33 (0.69)	0.266

The two groups did not significantly differ with respect to age, time since injury, and level of education. A significant difference at baseline was found between the two arms with respect to “physical fatigue”, as measured with a subscale of the DMFS. Therefore, an ANCOVA analysis was used to compare the groups (CBT-I + TAU group, TAU only group) on the performance on the subscale “physical fatigue” of the DMFS at T6 scores with T0 scores entered as covariate. No significant differences were found in other outcome measures between groups at baseline. Baseline data of the groups are presented in Table 3.

**Table 3:** Group by time interactions for the primary (PSQI) and secondary outcome measures for the CBT-I add-on intervention group and the treatment as usual group

Measure	Group	Pre-treatment		Post-treatment		Main effect of time			Time×group		
		*M*	*SD*	*M*	*SD*	*p* value	*F* (1,39)	*Cohen*’*s d*	*p* value	*F* (1,39)	*Cohen*’*s d*
Primary outcome measure											
PSQI	CBT-I + TAU	10.65	3.16	6.09	2.19	<0.001^**^	32.50	1.670	<0.001^**^	18.05	0.924
	TAU only	11.11	2.93	10.44	3.20
Secondary outcome measures											
HADS	CBT-I + TAU	14.57	7.17	10.78	5.80	0.001^**^	16.20	0.829	0.281	3.89	0.062
	TAU only	18.61	6.96	16.44	7.50
DMFS-I	CBT-I + TAU	37.00	9.86	33.00	9.17	0.049^*^	4.14	0.212	0.102	2.80	0.144
	TAU only	41.39	7.21	41.00	9.09
DMFS-II	CBT-I + TAU	31.00	5.43	29.35	5.31	0.281	1.20	0.062	0.108	2.71	0.139
	TAU only	31.44	5.44	31.78	5.36
DMFS-III	CBT-I + TAU	26.91	4.96	25.65	5.55	0.270	1.25	0.064	0.732	0.12	0.006
	TAU only	27.94	6.69	27.28	4.40
DMFS-IV	CBT-I + TAU	16.52	5.56	14.96	5.30	–	–	–	0.188	1.80	0.094
	TAU only	20.83	6.25	19.72	5.85
DMFS-V	CBT-I + TAU	16.78	2.98	14.44	4.30	0.039^*^	4.55	0.232	0.007^**^	8.06	0.413
	TAU only	16.39	4.19	16.72	4.74
DBAS-16	CBT-I + TAU	5.54	1.09	4.75	1.03	0.010^*^	7.28	0.372	0.010^*^	11.83	0.375
	TAU only	5.84	1.02	5.84	1.04

*Notes.* CBT-I + TAU group (N = 23). TAU only group (N = 18). PSQI = Pittsburgh Sleep Quality Index. HADS = Hospital Anxiety and Depression Scale. DMFS = Dutch Multifactor Fatigue Scale. DMFS-I=impact of fatigue. DMFS-II = mental fatigue. DMFS-III = signs and direct consequences of fatigue. DMFS-IV = physical fatigue. DMFS-V = coping with fatigue. DBAS-16 = Dysfunctional Beliefs and Attitudes about Sleep, Brief version. Cohen’s *d* is interpreted as small (≥0.2), moderate (≥0.5) and large (≥0.8) effects, respectively (Cohen, 2013). ^*^p < 0.05, ^**^p < 0.01, ^***^p < 0.001.

**Fig. 3:**
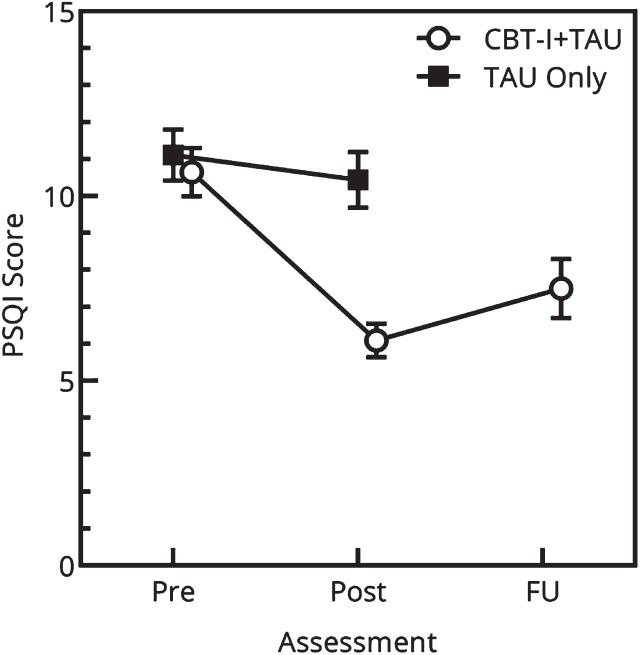
Results at baseline (T0) and post-treatment (T6) on the Pittsburgh Sleep Quality Index (PSQI) global score for the group receiving a cognitive-behavioural therapy-based add-on sleep intervention (CBT-I) in addition to rehabilitation treatment as usual (TAU) compared to a TAU Only group. Higher PSQI scores reflect a worse sleep quality and more sleep disturbances.

Before conducting the main analysis, relevant assumptions for Repeated Measure General Linear Model ANOVA (RM-ANOVA; normality, sphericity and independence) were checked and all assumptions were met. The group by time interactions for the outcome measures are shown in Table 3. The RM-ANOVA analysis showed a significant main effect of time in sleep quality within groups (*F* (1,39) = 32.502, *p* < 0.001). These results indicate that the groups changed (increased) in sleep quality over time. The RM-ANOVA analysis showed a significant interaction between group and time with a large effect size for the PSQI (*F*(1,39) = 18.047, *p* < 0.001). Participants in the CBT-I + TAU group showed a substantial decrease in PSQI scores, whereas no significant improvement was observed in the TAU only group. In other words, the short add-on therapy resulted in improved sleep quality in the CBT-I + TAU group relative to the TAU only group (Fig. 3).

The RM-ANOVA analyses conducted on the secondary outcome measures revealed a significant main effect of time for anxiety and depressive symptoms (*p* < 0.001), which indicate that the groups change (decreased) over time in both groups. Significant main effects of time were found on the DMFS subscale “impact of fatigue” (*p* = 0.049) and in beliefs and attitudes about sleep (measured with the DBAS-16) about sleep (*p* = 0.010). The impact of fatigue decreased and sleep related cognitions improved over time. There was a significant main effect of time in coping with fatigue (*p* = 0.039), which indicated that the groups changed in coping with fatigue (participants are able to take their fatigue into account and may adapt their daily functioning). However, this main effect of time could be attributed to the significant interaction effect for group over time on the DMFS subscale ‘coping’. The RM-ANOVA analyses showed a significant interaction with small effect sizes between group and time on dysfunctional beliefs and attitudes about sleep (DBAS-16) (*F*(1,39) = 7.299, *p* = 0.010) and the DMFS subscale “coping” (*F*(1,39) = 8.060, *p* = 0.007).

The ANCOVA did not show any significant difference between the two groups on T6 scores for physical fatigue when adjusted for T0 scores. In other words, there was no significant change in physical fatigue after the short add-on sleep therapy. Table 3 shows the mean scores on assessment of sleep quality at baseline, post-treatment and follow-up in the CBT-I + TAU group and TAU only group.

The RCI analyses showed that 11% of the patients (2 out of 18) receiving treatment as usual without the sleep intervention (TAU only group) reliably changed with respect to sleep quality during the six weeks waiting period, while 65% of the patients in the CBT-I + TAU group (15 out of 23) demonstrated reliable improvement in sleep quality when comparing scores before and after following the sleep intervention. Furthermore, at post-treatment, 35% of the participants (8 out of 23) had a PSQI score below the cut-off score for insomnia ≤5, whereas this was 0.1% (1 out of 18) at the post-waiting period.

In order to check whether the effects were maintained, a 3-month follow-up assessment was conducted, comparing the scores on the follow-up with the post-treatment performance. The 2-tailed, paired *t* test reflect that the effects of the short add-on sleep intervention on sleep quality (t(17) = – 2,07, *p* = 0.054), sleep-related cognitions (t(18) = – 1.56, *p* = 0.135) and coping with fatigue (t(19) = – 0.96, *p* = 0.351) were maintained at follow-up compared to posttreatment (Table 4).

**Table 4:** Paired sample *t*-tests for the long-term follow up assessment for the primary and secondary outcome measures for the CBT-I + TAU group

Measure	Follow-up			Paired differences M (SD)	*t*-test
	N	M	SD
PSQI	18	7.50	3.37	–0.12 (2.51)	*t*(17) = – 2.07, *p* < 0.054
HADS	20	13.15	6.75	–2.55 (3.53)	*t*(19) = – 3.23, *p* < 0.004^**^
DMFS-I	19	37.63	9.98	–3.16 (8.11)	*t*(18) = – 1.70, *p* < 0.107
DMFS-II	19	31.32	6.53	–1.11 (5.50)	*t*(18) = – 0.88, *p* < 0.392
DMFS-III	19	27.32	5.26	–1.32 (2.71)	*t*(18) = – 2.12, *p* < 0.048^*^
DMFS-IV	19	16.16	5.17	–0.42 (4.26)	*t*(18) = – 0.43, *p* < 0.672
DMFS-V	19	15.74	3.83	–1.32 (3.67)	*t*(18) = – 1.56, *p* < 0.135
DBAS-16	20	4.86	1.01	–0.16 (0.76)	*t*(19) = – 0.96, *p* < 0.351

*Notes.* PSQI = Pittsburgh Sleep Quality Index. HADS = Hospital Anxiety and Depression Scale. DMFS = Dutch Multifactor Fatigue Scale. DBAS-16 = Dysfunctional Beliefs and Attitudes about Sleep, Brief version.

## 4 Discussion

This randomized controlled trial (RCT) investigated the effectiveness of a short add-on CBT-I-based therapy for sleep disturbances in individuals with ABI in addition to rehabilitation treatment as usual. The CBT-I + TAU group improved more on self-reported sleep quality, dysfunctional beliefs and attitudes about sleep and partially on fatigue relative to the TAU only group. Specifically, the CBT-I + TAU group significantly improved on sleep quality with large effect sizes over time relative to the TAU only group. Furthermore, sleep-related cognitions significantly improved in the CBT-I + TAU group, with small effect sizes (ES = 0.375), as compared to the TAU only group, and participants in the CBT-I + TAU group were better able to cope with fatigue. No significant treatment effects were found regarding impact, consequences or symptoms of fatigue, mental and physical fatigue, or in anxiety and depressive symptoms. Our results also showed that the post-treatment improvement (e.g. sleep quality, sleep related cognitions and coping with fatigue) were maintained at 3-month follow-up in the group that received the add-on treatment.

These results corroborate and extend previous findings showing that CBT is effective in improving sleep in individuals with ABI (Herron et al., 2018; Nguyen et al., 2019; Ouellet & Morin, 2004, 2007; Pilon et al., 2021). The present study seems to have comparable efficacy for ABI-related sleep disturbances compared to previous CBT-I studies. Moreover, the reported effect sizes for the short CBT-I intervention over time were in roughly the same range as the more conventional CBT-I recommended treatments for sleep disturbances (Herron et al., 2018; Ouellet & Morin, 2007). Our findings also show that a short add-on sleep therapy based on CBT-I components is already effective, which has the advantage of being more accessible and scalable in rehabilitation care compared to traditional CBT-I. That is, the short add-on sleep therapy is delivered in a shorter amount of time (4 sessions in a period of 6 weeks compared to 6–8 sessions). Also, the intervention can be provided by trained cognitive rehabilitation therapists (e.g., occupational therapists), which makes this not only a cost-effective intervention but also more widely available, as experienced and certified CBT professionals are scarce in the field of rehabilitation.

### 4.1 Limitations and future directions

Although our findings provide valuable information on treatment efficacy of a short CBT-I treatment for sleep disturbances in the ABI population, some limitations should be noted. First, the present study aimed to examine the effectiveness of an add-on sleep intervention in individuals with ABI. The Consolidated Standards of Reporting Trials (CONSORT) statement recommends that a RCT study reports detailed information about adherence to treatment (Boutron et al., 2017). The present study did not monitor adherence to the study-specific module, therefore it is not possible to evaluate whether the participants received their allocated treatment exactly as intended. Nevertheless, to improve adherence, the add-on sleep intervention was delivered by a limited number (i.e., 3) of trained therapists who were all supervised by one of the developers of the protocol. A second limitation of the study is the high dropout rate (24%). Although there were no clear indications that participants dropped out due to the add-on sleep intervention, it might have affected the results. Furthermore, the COVID-19 pandemic lockdowns that took place in 2020 required protocol adjustments (e.g. online sessions through video calls), which did not only affect this intervention, but also the rehabilitation care as usual, which may have led to higher drop outs rates and/or smaller effect sizes of the intervention. Fourth, the present study did not compare the short add-on therapy with the standard CBT-I intervention. Future research should compare the short add-on sleep intervention to a regular CBT-I intervention.

## 5 Conclusions

Sleep disturbances are common following ABI and have a negative impact on emotional well-being and cognitive functioning. The present study shows that a short CBT-I based sleep intervention produced significant improvements in sleep quality and fatigue in people with ABI in addition to rehabilitation treatment as usual. Furthermore, participants who received the sleep therapy were better able to cope with fatigue compared to the TAU only group. No significant improvements were observed regarding impact and consequences of fatigue, symptoms of physical and mental fatigue, nor in anxiety and depressive symptoms. It is also important to note that some potentially burdensome elements in the add-on sleep intervention, such as stimulus control and sleep restriction, did not increase fatigue during treatment. This study shows that psychoeducation combined with behavioural recommendations to change sleep behaviour seems to be sufficient and confirms the shortening of the regular CBT-I protocol in this ABI population. The application of this short add-on sleep intervention could be implemented in neuropsychological rehabilitation settings.
